# Impact of intermittent high-dose radon exposures on lung epithelial cells: proteomic analysis and biomarker identification

**DOI:** 10.1093/jrr/rraf010

**Published:** 2025-03-15

**Authors:** Phawinee Subsomwong, Chutima Kranrod, Yuna Sakai, Krisana Asano, Akio Nakane, Shinji Tokonami

**Affiliations:** Department of Microbiology and Immunology, Hirosaki University Graduate School of Medicine, 5 Zaifu-cho, Hirosaki, Aomori 036-8562, Japan; Institute of Radiation Emergency Medicine, Hirosaki University, 66-1 Hon-cho, Hirosaki, Aomori 036-8564, Japan; Institute of Radiation Emergency Medicine, Hirosaki University, 66-1 Hon-cho, Hirosaki, Aomori 036-8564, Japan; Department of Microbiology and Immunology, Hirosaki University Graduate School of Medicine, 5 Zaifu-cho, Hirosaki, Aomori 036-8562, Japan; Department of Biopolymer and Health Science, Hirosaki University Graduate School of Medicine, 5 Zaifu-cho, Hirosaki, Aomori 036-8562, Japan; Institute of Radiation Emergency Medicine, Hirosaki University, 66-1 Hon-cho, Hirosaki, Aomori 036-8564, Japan

**Keywords:** radon exposure, cytotoxicity, proteome, lung cancer, biomarkers

## Abstract

Lung cancer is the most prevalent cancer worldwide, and radon exposure is ranked as the second risk factor after cigarette smoking. It has been reported that radon induces deoxyribonucleic acid damage and oxidative stress in cells. However, the protein profile and potential biomarkers for early detection of radon-induced lung cancer remain unknown. In this study, we aimed to investigate the effects of intermittent high-dose radon exposure on lung epithelial cells, analyze protein profiles and identify potential biomarkers for diagnosis of radon-related lung cancer. Human lung epithelial cells (A549) were exposed to radon (1000 Bq/m^3^) for 30 min daily for 7 days. Cell viability was measured using the WST-1 assay, and liquid chromatography–mass spectrometry proteomic analysis was performed. Differentially expressed proteins and gene ontology (GO) enrichment were analyzed. Our findings showed that intermittent high-radon exposure reduced A549 cell viability over time. Proteomic analysis identified proteins associated with stressed-induced apoptosis, mitochondrial adaptation, nuclear integrity and lysosomal degradation. These proteins are related to catabolism, stress response, gene expression and metabolic processes in the biological process of GO analysis. We highlighted specific proteins, including AKR1B1, CDK2, DAPK1, PRDX1 and ALHD2 with potential as biomarkers for radon-related lung cancer. In summary, intermittent high-dose radon exposure affects cellular adaptions of lung epithelial cells including stress-induced apoptosis, mitochondrial dysfunctions and immune regulation. The identified proteins may serve as diagnostic biomarkers or therapeutic targets for radon-related lung cancer.

## INTRODUCTION

Lung cancer is one of the leading causes of cancer-related deaths globally with exposure to carcinogenic agents including tobacco smoke, pollution and radon [1, 2]. Radon is a naturally occurring radioactive gas that is produced from the decay of uranium. This gas is colorless, odorless and tasteless making it difficult to detect [3]. The World Health Organization (WHO) classified radon as the second leading cause of lung cancer in non-smokers, especially in regions with high radon concentrations. WHO recommends an indoor radon concentration range of 100–300 Bq/m^3^ for residential spaces [4]. Furthermore, the high background radiation locations and radon spas comprise natural springs, thermal pools or enclosed environments in the presence of radium-rich rocks, all of which are found in areas with extremely high radon concentrations; measured values can approach hundreds of Bq/m^3^ [5, 6]. These high exposures have raised some health issues not just for the individuals who live in or are treated in spas but also for workers at the spas. Additionally, non-smokers exposed to radon at the level of 800–1000 Bq/m^3^ have an approximately 6% lifetime chance of developing lung cancer [7]. Also, epidemiologic studies of radon and lung cancer in North America, Europe and China pooling data shown that the excess risk of lung cancer per 100 Bq/m^3^ of radon gas is estimated to be around 10% (range 8–16%) for both ever-smokers and ex-smokers [8].

Radon gas releases alpha particles through the radioactive decay. When inhaled, its progeny can deposit along the lung epithelial cells leading to tissue damage over time. Radon exposure induces deoxyribonucleic acid (DNA), damage and impairs DNA repair mechanisms, which can contribute to mutations that disrupt cell cycle regulation, promote genomic instability and activate oncogenes [9, 10]. Additionally, radon induces oxidative stress by generating reactive oxygen species that can damage cellular components (CCs). Other effects of radon exposure have been reported, including mitochondrial dysfunction, apoptosis induction and inflammatory responses [11]. However, few studies have focused on the protein profile and potential biomarkers for early diagnosis of lung cancer caused by radon.

In this study, we aim to investigate the effects of intermittent high-dose radon exposure on lung epithelial cell viability and protein profiles using proteomic analysis. There has been no research on exposing epithelial cells *in vitro* to intermediate radon concentrations (1000 Bq/m^3^). We identified differentially expressed proteins (DEPs) and performed gene ontology (GO) analysis to uncover the cellular pathway affected by radon. In addition, we identified potential biomarkers and therapeutic targets specific to radon-related lung cancer. We found that intermittent high-dose radon exposure affects cell viability and cellular functions. Our study provides the candidate proteins that may serve as the specific biomarkers for radon-related lung cancer.

## MATERIALS AND METHODS

### Cell culture

Human lung epithelial (A549) cell line was obtained from the American Type Culture Collection (CCL-185). The A549 cells were cultured in Ham’s F-12 K medium (FUJIFILM Wako Pure Chemical Co., Osaka, Japan) supplemented with 10% Fetal Calf Serum (FCS; JRH Biosciences, Lenexa, KS), 0.03% L-glutamine (FUJIFILM Wako) and antibiotic-antifungal combination agent (Gibco™ Antibiotic-Antimycotic; Thermo Fisher Scientific, Waltham, MA).

### Radon exposure

A549 cells were seeded in 96-well plates (1 × 10^5^ cells/well) for cytotoxicity assay and 6-well plates (3 × 10^5^ cells/well) for proteomic analysis. The plates were incubated at 37°C under 5% CO_2_ for 24 h, and the culture medium was replaced with fresh medium before the plate was placed in an in-house radon gas inhalation chamber (Hirosaki University, Aomori, Japan). Natural uranium rocks were used as a radon source in the radon exposure system [12]. Radon gas was injected into an acrylic inhalation chamber of 0.012 m^3^ by an air pump flow rate of 20 L/min through an acrylic chamber of 0.54 m^3^ for stable radon gas concentration. The cells were exposed to radon 1000 Bq/m^3^ for 30 min per day and intermitted daily for seven days. The estimate of absorbed dose to cells in this research was approximately 12.6 μGy. Control cells were cultured under the same conditions without radon exposure.

### Cytotoxic assay

WST-1 Cell Proliferation Reagent (Sigma-Aldrich, St. Louis, MO) was used to detect cell viability after radon exposure. Cell viability was evaluated 24 h after radon exposure on days 1, 3, 5 and 7. Briefly, 10 μL of WST-1 reagent was added directly to each well and incubated at 37°C under 5% CO_2_ until the color development. The optical density at 450 nm was measured using a microplate reader (MULTISKAN Sky, Thermo Fisher Scientific). The absorbance values were subtracted with those of cell medium without A549 cells as background control. The cell viability of A549 cells without radon exposure was set as 100%.

### Sample preparation for proteomic analysis

On day 7 of radon exposure, A549 cells were washed three times with ice-cold 1x phosphate-buffered saline (PBS) supplemented with a protease inhibitor (Roche Diagnostics GmbH, Mannheim, Germany), followed by two washes with PBS. The cells were lysed in lysis buffer (50 mM Tris pH 8.0, 150 mM NaCl, 1% NP40 and 0.5% sodium deoxycholate) using a shaker (EYELA CM-1000, Tokyo Rikakikai Co. Ltd., Tokyo, Japan) for 15 min at room temperature. Cell lysates were then stored at −80°C until further proteomic analysis by liquid chromatography with tandem mass spectrometry (LC–MS/MS). The protein concentration of cell lysates was measured using Bradford protein assay using Bio-Rad Protein Dye Reagent Concentrate (Bio-Rad Laboratories, Inc., Hercules, CA).

### Proteomic analysis

Proteins were identified by LC–MS/MS using Triple TOF 6600 instrument (AB Sciex, Tokyo, Japan) at the Scientific Research Facility Center, Japan. The spectra were matched to the proteins of the A549 cell database using the ProteinPilot software (v5.0.1, AB Sciex). The resulting file was imported into PeakView (v2.2.0, AB Sciex) as a library, and peaks from SWATH-MS runs were extracted with a false discovery rate of <1%. The data were then exported to the MarkerView software program (v1.3.0.1; AB Sciex), and the peak area of each protein was normalized relative to the total peak areas for all detected proteins. The data were subsequently prepared for downstream analyses, including DEPs. A volcano plot of DEPs was generated using GraphPad Prism 10 software (v10.0; GraphPad Software Inc., La Jolla, CA). Functional analysis of GO terms, DEPs were analyzed and visualized using Cytoscape software (v.3.10.1) with the ClueGO/CluePedia plugin. GO terms were categorized into biological process (BP), CC and molecular function (MF) in a hierarchical structure as described previously [13].

### Statistical analysis

The statistical analyses were performed using GraphPad Prism and the method mentioned in each figure legend. The *P*-value was <0.05 was considered statistically significant.

## RESULTS

### Radon exposure affects the cell viability of lung epithelial cells

**Fig. 1:**
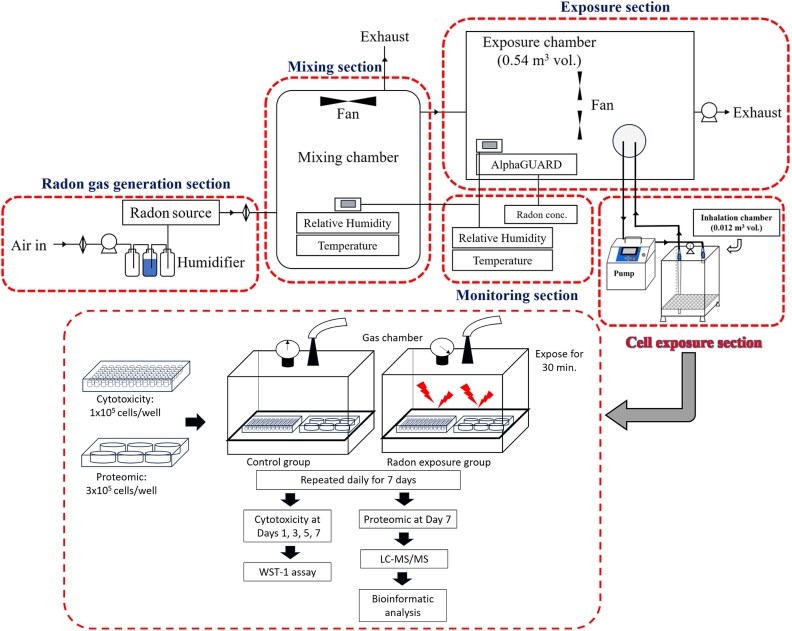
Schematic overview of the experimental protocol. A549 cells were seeded in 96-well and 6-well plates for cytotoxicity and proteomic analyses, respectively. The cells were exposed to radon (1000 Bq/m^3^) for 30 min daily for 7 days. Control cells were cultured without radon exposure. Cytotoxicity was observed on days 1, 3, 5 and 7 using WST-1 assay. Protein identification was conducted on day 7 using LC–MS/MS and bioinformatic analyses.

**Fig. 2:**
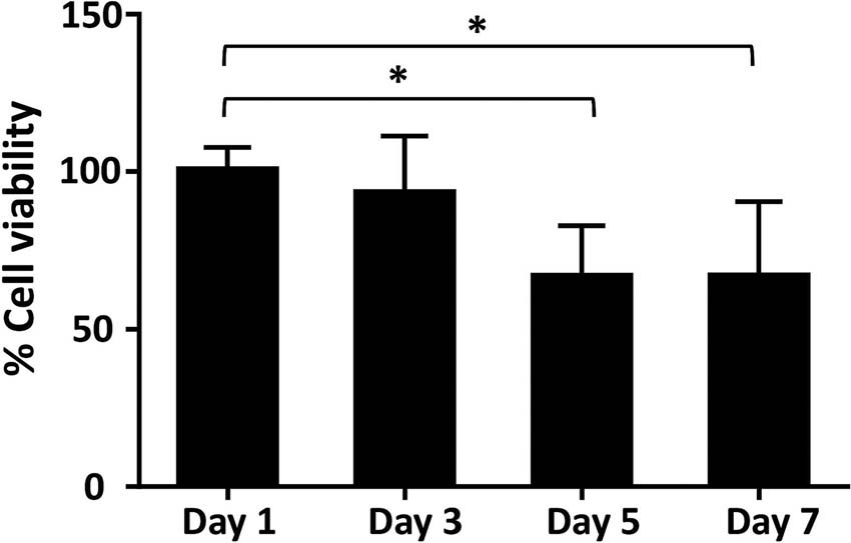
Effect of radon exposure on the viability of lung epithelial cells. A549 cells (1 × 10^5^ cells/well) were seeded in 96-well plates, and cell viability was observed 24 h after radon exposure on days 1, 3, 5 and 7 using WST-1 assay. Absorbance was measured at 450 nm. The cell viability of A549 cells without radon exposure was set as 100% (*n* = 6 from two independent experiments). Statistical analysis was performed using the Kruskal–Wallis H-test by Dunn’s multiple comparison test (* *P* < 0.05).

To investigate the cytotoxic effect of high-dose radon on lung epithelial cells, the A549 cells were exposed with or without radon for 30 min, repeated daily for 7 days (Fig. 1). Cell viability was examined using WST-1 assay on days 1, 3, 5 and 7. Radon exposure reduced the A549 cell viability in a time-dependent manner (Fig. 2). Cell viability on days 5 and 7 was significantly decreased by approximately 30% compared to the cells at day 1 (*P* < 0.05). This suggests that intermittent high-dose radon exposure over time caused the cytotoxicity.

### Differential expression proteins of A549 cells after radon exposure

To investigate the effect of intermitted radon exposure on lung epithelial cells, the cells were exposed with or without radon for 30 min daily for 7 days. After exposure, the cells were collected and analyzed for their proteomic profiles using LC–MS/MS (Fig. 1).

**Fig. 3:**
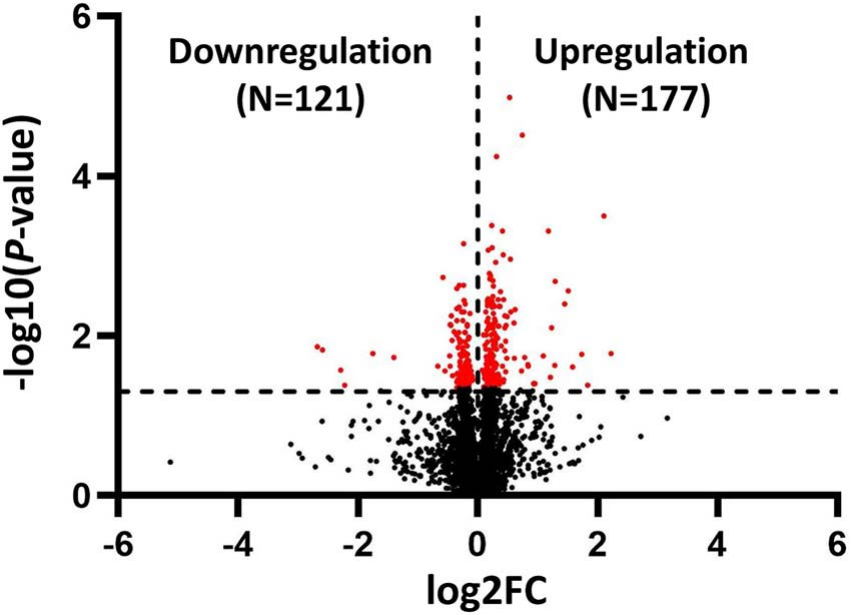
Volcano plot of differential expression proteins in radon-exposed A549 cells and unexposed controls. Black dots represent proteins without significant differential expression, while red dots indicate proteins with significant changes [cutoff: -log10 (*P*-value) > 1.3 and log2FC > 0 or log2FC < 0].

A total of 4486 proteins were identified, and DEPs between radon-exposed A549 cells and unexposed controls were determined. A volcano plot of DEPs was generated using GraphPad Prism 10 software as shown in Fig. 3. A total of 298 DEPs were significantly different [−log10 (*P*-value) > 1.3 and log2FC > 0 or log2FC < 0]. One hundred and seventy-seven proteins were upregulated, and 121 proteins were downregulated in radon-exposed cells (Table S1). Localization of these proteins is predominantly in the cytoplasm (41%), nucleus (20%), cell membrane (10%) and mitochondrion (8%) as shown in Fig. S1 and Table S1. The top five upregulated proteins were interferon regulatory factor 9 (IRF9), v-type proton ATPase subunit B (ATP6V1B1), tax1-binding protein 3 (TAX1BP3), complex III assembly factor (LYRM7), and protein argonaute-1 (AGO1). These proteins counteract the effects of radon exposure, including immune activation, stress-induced apoptosis regulation, enhanced degradation of damaged components, and mitochondrial adaptation. The top five downregulated proteins were protein unc-93 homolog B1 (UNC93B1), transmembrane protein 256 (TMEM256), nuclear envelope phosphatase-regulatory subunit 1(CNEP1R1), β-glucuronidase (GUSB) and DnaJ homolog subfamily C member 17 (DNAJC17). These results indicate that radon exposure disrupted key cellular processes, such as membrane signaling, nuclear integrity, lysosomal degradation and protein homeostasis, which can lead to cell stress, cell damage and cell death.

### GO analysis of DEPs

**Fig. 4:**
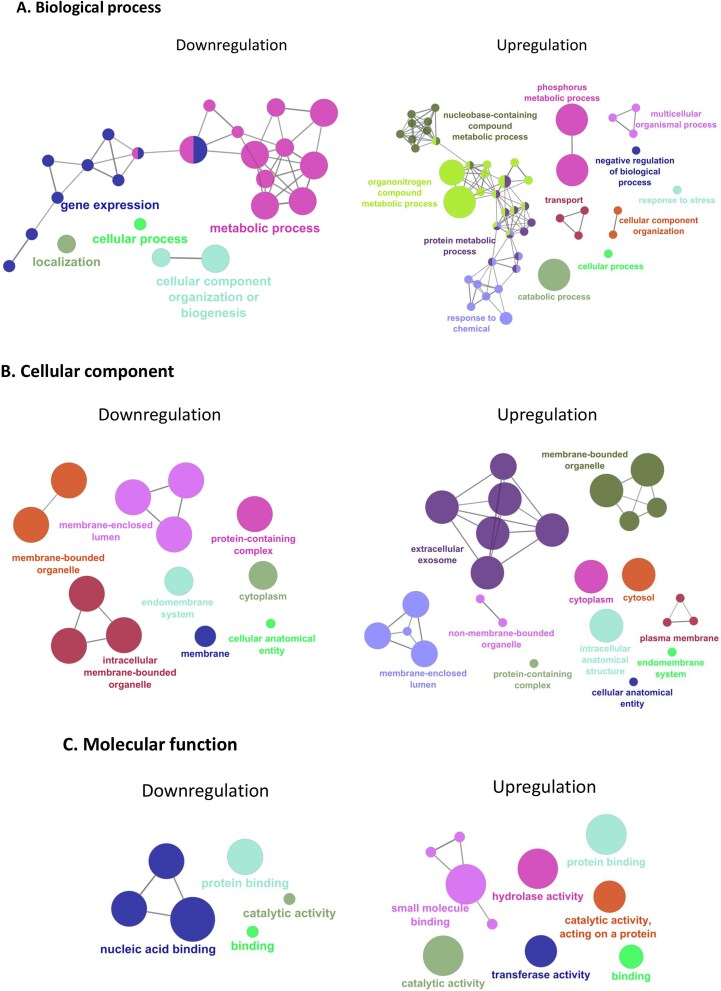
Gene Ontology analysis was visualized using the ClueGO/CluePedia in Cytoscape. (A) BP, (B) CC and (C) MF categories for DEPs between radon-exposed A549 cells and unexposed controls. The node colors represent specific functional classes associated with the enrichment analysis. Distinct colors indicate various BP, CC and MF pathways involved in the enrichment analysis of identified DEPs.

To understand the overview of these upregulated and downregulated proteins of intermittent high-dose radon exposure A549 cells, we performed a detailed analysis of DEPs. The Cytoscape plugin ClueGO/CluePedia was used to study the GO enrichment of the DEPs in terms of BP, CC and MF shown in Fig. 4 and Table 1. The BP analysis revealed that processes such as catabolic process, response to stress, phosphorus metabolic process, CC organization and response to chemical were upregulated, while localization, gene expression and metabolic process were downregulated. These proteins contribute to cellular stress and cell death (Fig. 4A, Table 1). Regarding CC, upregulated and downregulated proteins were observed in the intracellular anatomical structure, cytosol, non-membrane-bound organelles, extracellular exosome, intracellular membrane-bounded organelle and others (Fig. 4B, Table 1). In MF terms, transferase activity, hydrolase activity, catalytic activity acting on a protein, small molecule binding and nucleic acid binding were affected (Fig. 4C, Table 1). Taken together, the upregulation of catabolism, stress response and transferase/hydrolase activity suggest that radon damages cells by degrading CCs, reorganizing the internal structure and affecting stress response pathways. Additionally, the downregulation of gene expression, nucleic acid binding and intracellular membrane-bound organelles indicate that the radon-exposed cells lost their ability to adapt, repair and maintain their functions resulting in cellular damage and reduced viability.

**Table 1:** GO enrichment categories of radon-exposed A549 cells

GO BP	ID	Term
Up/downregulation		
Upregulation	**GO:0009056**	**Catabolic process**
	GO:0009987	Cellular process
	**GO:0006950**	**Response to stress**
	**GO:0048519**	**Negative regulation of BP**
	**GO:0006793**	**Phosphorus metabolic process**
	**GO:0016043**	**CC organization**
	**GO:0032501**	**Multicellular organismal process**
	**GO:0006810**	**Transport**
	**GO:0006139**	**Nucleobase-containing compound metabolic process**
	**GO:0042221**	**Response to chemical**
	**GO:0019538**	**Protein metabolic process**
	**GO:1901564**	**Organonitrogen compound metabolic process**
Downregulation	**GO:0051179**	**Localization**
	GO:0009987	Cellular process
	**GO:0071840**	**CC organization or biogenesis**
	**GO:0010467**	**Gene expression**
	**GO:0008152**	**Metabolic process**
GO CC	ID	Term
Up/downregulation		
Upregulation	GO:0012505	Endomembrane system
	GO:0032991	Protein-containing complex
	**GO:0005622**	**Intracellular anatomical structure**
	GO:0110165	Cellular anatomical entity
	GO:0005737	Cytoplasm
	**GO:0005829**	**Cytosol**
	**GO:0043228**	**Non-membrane-bounded organelle**
	**GO:0005886**	**Plasma membrane**
	GO:0043227	Membrane-bounded organelle
	GO:0031974	Membrane-enclosed lumen
	**GO:0070062**	**Extracellular exosome**
Downregulation	GO:0005737	Cytoplasm
	GO:0110165	Cellular anatomical entity
	GO:0012505	Endomembrane system
	**GO:0016020**	**Membrane**
	GO:0032991	Protein-containing complex
	GO:0043227	Membrane-bounded organelle
	GO:0031974	Membrane-enclosed lumen
	**GO:0043231**	**Intracellular membrane-bounded organelle**
GO MF	ID	Term
Up/downregulation		
Upregulation	GO:0003824	Catalytic activity
	GO:0005488	Binding
	GO:0005515	Protein binding
	**GO:0016740**	**Transferase activity**
	**GO:0016787**	**Hydrolase activity**
	**GO:0140096**	**Catalytic activity, acting on a protein**
	**GO:0036094**	**Small molecule binding**
Downregulation	GO:0003824	Catalytic activity
	GO:0005488	Binding
	GO:0005515	Protein binding
	**GO:0003676**	**Nucleic acid binding**

Bold: GO terms observed only in upregulated or downregulated proteins.

### DEPs-related lung cancer

Regarding DEPs in Table S1, the top five upregulated and downregulated proteins could be used as the candidate markers for radon-related lung cancer. However, some of the DEPs are not only found in lung cancer but also relevant in the prognosis of other cancers. For example, IRF9 is involved in the interferon signaling pathway and regulating immune response. It has been reported as a potential biomarker or drug target in lung, breast and melanoma [14–16]. Other DEPs including aldo-keto reductase family 1 member B1 (AKR1B1), cyclin-dependent kinase 2 (CDK2), death associate protein kinase-1 (DAPK1), peroxiredoxin-1 (PRDX1) and aldehyde dehydrogenase mitochondrial (ALDH2) are associated with lung cancer [17–21]. AKR1B1 has been reported overexpressed in cancer and is associated with inflammatory mediators including nuclear factor kappa-light-chain enhancer of activated B cells (NFκB), as well as cell cycle mediators such as cyclin-dependent kinases (CDKs), and survival proteins [17]. CDK2, is identified in the small-cell lung cancer pathway by the KEGG pathway (PATH:ko05222), and it plays a role in the cell cycle. CDK2 is activated in the late phase of the cell cycle by binding to cyclins E (Cyclin E/CDK2 complex), this activation promotes the cell’s progression from the G1 phase to the S phase where the DNA replications occur [22]. DAPK1, found in the cancer pathway (hsa05200), is a part of the Ser/Thr kinase family and is an important regulator of cell death and autophagy [23]. PRDX1 has been reported to regulate cell growth, differentiation and apoptosis [20]. ALDH2, a member of the ALDH superfamily, plays a role in detoxifying aldehydes and against oxidative stress [24]. In this study, the levels of AKR1B1, CDK2, DAPK1 and PRDX1 were higher in radon-exposed A549 cells compared to unexposed controls, while the ALDH2 level was lower in radon-exposed A549 cells, as shown in bold in Table S1. Taken together, these five proteins have potential to be used as biomarkers for early-stage detection or therapeutic targets for radon-related lung cancer. However, their broader implications across other types of cancer should be considered.

## DISCUSSION

Lung cancer is one of the highest mortality rates among cancers worldwide and is associated with environmental and lifestyle factors, including exposure to carcinogenic agents and smoke [25, 26]. Among non-smoking populations, radon exposure is considered a major risk factor, especially in the regions with naturally high radon levels [27]. In this study, we investigated the impact of intermittent high-dose radon exposure on lung epithelial cell viability, protein profiles, protein functions and expression levels. Additionally, we identified candidate proteins that could potentially serve as biomarkers for diagnosing radon-associated lung cancer. Our finding revealed that intermitted radon exposure at 1000 Bq/m^3^ for 30 min for a week led to a 30% decrease in A549 cell viability by days 5 and 7. This suggests that prolonged radon exposure might affect the functions and critical proteins necessary for lung cell viability. The meta-analysis revealed that an increase in residential radon levels of 100 Bq/m^3^ was associated with a higher risk of lung cancer, including small-cell lung carcinoma and adenocarcinoma [28]. Loiselle *et al.* reported that a low concentration of radon (38 Bq/m^3^) exposure to lung cell lines for a week could change their gene expression, particularly the AKR1C3 gene. They concluded that prolonged and consistent exposure to low concentrations of radon exposure could enhance the risk of carcinogenesis [29]. Likewise, Chen *et al.* demonstrated that repeated radon exposure (20 000 Bq/m^3^ for 20 min each time every 3 days) affected cell adhesion, proliferation and invasion in lung cells [30]. Therefore, in our study, we investigated changes in protein expression in A549 cells after being exposed to intermittent radon for a week of proteomic analysis.

Protein identification and GO analysis of DEPs in radon-exposed cells revealed several proteins associated with cell function and stress response. Specifically, IRF9, ATP6V1B1, TAX1BP3, LYRM7 and AGO1 were upregulated, while UNC93B1, TMEM256, CNEP1R, GUSB and DNAJC17 were downregulated in A549 cells exposed to radon. These proteins are related to stressed-induced apoptosis, mitochondrial adaptation, membrane signaling, nuclear integrity and lysosomal degradation. GO analysis of DEPs indicated upregulation in catabolic and stress response pathways, along with downregulation in gene expression, and metabolic processes within BP. Additionally, radon exposure disrupted cellular functions, including transferase, hydrolase and catalytic activities. These protein dysfunctions might contribute to impaired cellular processes and membrane stability causing cellular stress and reduced viability. Many studies have shown that radon exposure induces cytotoxic effects in lung cells through several mechanisms, including DNA damage, oxidative stress, mitochondrial dysfunction, inflammatory responses, apoptosis and cell death pathways [9–11]. Radon exposure causes DNA damage and leads to different types of chromosome damage, even at very low doses [31]. Both *in vitro* and *in vivo* models demonstrated that long-term radon exposure causes cellular and lung tissue injury via oxidative stress, mitochondrial dysfunction, mitophagy and autophagy [11, 32].

Early-stage cancer detection significantly improves quality of life and survival rates. The DEPs in A549 cells exposed to high-dose radon are listed in Table S1. Among them, AKR1B1, CDK2, DAPK1 and PRDX1 were upregulated, while ALDH2 was downregulated. These proteins show potential as biomarkers for radon-related lung cancer. AKR1B1, a promising diagnostic biomarker and its inhibition has been shown to enhance the sensitivity of tumor cells to anti-cancer drugs [17]. A meta-analysis of a case–control study reported that an increase in residential radon levels by 100 Bq/m^3^ raised the risk of lung cancer, small-cell lung carcinoma and adenocarcinoma by 11%, 19% and 13%, respectively [28]. CDK2 is activated during the late phase of the cell cycle by binding to cyclins E, promoting progression through the G1/S phase where DNA replications occur [22]. Additionally, CDK2 expression is strongly associated with immune responses in cancer and prognostic value in lung adenocarcinoma [18]. DAPK1 has been implicated in non-small cell lung cancer (NSCLC), where its overexpression under conditions of oxygen and glucose deprivation induces excessive autophagy and apoptosis in A549 cells [19]. A study reported that both Prx-1 (PRDX1) antibody and antigen were positive in serum from NSCLC patients but none in the serum control from healthy subjects [33]. Conversely, the downregulation of ALDH2 is associated with poor prognosis in lung adenocarcinoma (LUAD). ALDH2 detoxifies acetaldehyde (ACE) to non-toxic acetic acid, and its repression leads to ACE accumulation, which is linked to increase in DNA damage [21]. Recent study has shown significant lower ALDH2 gene and protein expression in LUAD tissues compared to normal tissues. This downregulation affects DNA methylation and disrupts stem cell-related pathways [34].

The limitations of our study are (i) we investigated the effect of intermittent high-dose radon exposure on a single type of lung cell line (A549 cell) which may not represent all lung cell types. Further studies using additional cell lines or primary cells are necessary to validate our findings. (ii) Some candidate DEPs identified in this study are known markers for various cancers. For instance, IRF9 has been reported as a promising therapeutic and biomarker target for skin malignancy and breast cancer [15, 16]. Therefore, further validation of candidate proteins and their specificity to radon exposure-related experiments are required in future studies.

In conclusion, this study demonstrated that intermittent high-dose radon exposure significantly impacts lung epithelial cell viability by activating the cytotoxic effect and reducing cell survival rates over the exposure period. The proteins related to cellular adaptations including stress-induced apoptosis, mitochondrial dysfunctions and immune regulation were identified in radon-exposed lung cells. The proteins such as AKR1B1, CDK2, DAPK1, PRDX1 and ALDH2 show potential as biomarkers or therapeutic targets for radon-related lung cancer after further validation.

## Supplementary Material

Table_S1_rraf010

Fig_S1_rraf010

## Data Availability

The proteomic data of A549 cells unexposed and exposed to radon have been deposited in Proteome Xchange and jPost Repository under accession numbers PXD058364 and JPST003489. Data and supporting materials associated with this study will be available from the corresponding author on reasonable request.
