# Supplementary material for: Impact of intermittent high-dose radon exposures on lung epithelial cells: proteomic analysis and biomarker identification
Source: J Radiat Res. 2025 Mar 15;66(2):107–14. doi: 10.1093/jrr/rraf010 (PMC11932336; doi:10.1093/jrr/rraf010)
Supplement: Table_S1_rraf010 [file table_s1_rraf010.docx]

| **Table S1.** The proteins significantly upregulation and downregulation in radon-exposure A549 cells. | | | | | | |
| --- | --- | --- | --- | --- | --- | --- |
| **Upregulation** | | | | | | |
| No. | Genes | Proteins | Control | Radon | *P* -value | Location |
| 1 | *IRF9* | Interferon regulatory factor 9 | 0.7 | 3.0 | 0.017 | Cytoplasm |
| 2 | *ATP6V1B1* | V-type proton ATPase subunit B, kidney isoform | 4.2 | 17.8 | 0.000 | Others |
| 3 | *TAX1BP3* | Tax1-binding protein 3 | 1.3 | 4.5 | 0.042 | Cytoplasm |
| 4 | *LYRM7* | Complex III assembly factor LYRM7 | 1.7 | 5.7 | 0.017 | Mitochondrion |
| 5 | *AGO1* | Protein argonaute-1 | 2.9 | 8.7 | 0.025 | Cytoplasm |
| 6 | *DNHD1* | Dynein heavy chain domain-containing protein 1 | 12.0 | 34.2 | 0.003 | Others |
| 7 | *LRRFIP2* | Leucine-rich repeat flightless-interacting protein 2 | 1.2 | 3.2 | 0.004 | Unknown |
| **8** | ***AKR1B1*** | **Aldo-keto reductase family 1 member B1** | **1070.7** | **2616.1** | **0.002** | **Cytoplasm** |
| **9** | ***CDK2*** | **Cyclin-dependent kinase 2** | **1.7** | **4.1** | **0.023** | **Cytoplasm** |
| 10 | *AGFG2* | Arf-GAP domain and FG repeat-containing protein 2 | 4.0 | 9.4 | 0.008 | Cytoplasm |
| **11** | ***DAPK1*** | **Death-associated protein kinase 1** | **2.9** | **6.6** | **0.033** | **Cytoplasm** |
| 12 | *FBXO2* | F-box only protein 2 | 46.4 | 105.3 | 0.000 | Cytoplasm |
| 13 | *ATXN3* | Ataxin-3 | 2.4 | 5.0 | 0.018 | Nucleus |
| 14 | *INPP4B* | Inositol polyphosphate 4-phosphatase type II | 3.1 | 6.0 | 0.039 | Cytoplasm |
| 15 | *CAVIN3* | Caveolae-associated protein 3 | 3.1 | 5.9 | 0.040 | Cytoplasm |
| 16 | *ARF3* | ADP-ribosylation factor 3 | 8.0 | 15.2 | 0.040 | Golgi apparatus |
| 17 | *GLUL* | Glutamine synthetase | 4.3 | 7.7 | 0.024 | Cytoplasm |
| 18 | *TBC1D4* | TBC1 domain family member 4 | 8.0 | 14.3 | 0.023 | Cytoplasm |
| 19 | *GFPT2* | Glutamine--fructose-6-phosphate aminotransferase [isomerizing] 2 | 4.4 | 7.6 | 0.019 | Cytosol |
| 20 | *GLS* | Glutaminase kidney isoform, mitochondrial | 38.8 | 64.9 | 0.000 | Mitochondrion |
| 21 | *STEAP3* | Metalloreductase STEAP3 | 5.0 | 8.2 | 0.028 | Endosome |
| 22 | *FHL1* | Four and a half LIM domains protein 1 | 5.5 | 8.5 | 0.005 | Cytoplasm |
| 23 | *CD70* | CD70 antigen | 10.3 | 15.6 | 0.019 | Cell membrane |
| 24 | *CDA* | Cytidine deaminase | 15.6 | 23.8 | 0.007 | Cytosol |
| 25 | *DDB2* | DNA damage-binding protein 2 | 12.2 | 17.8 | 0.005 | Nucleus |
| 26 | *OGFR* | Opioid growth factor receptor | 12.4 | 18.1 | 0.001 | Cytoplasm |
| 27 | *PC* | Pyruvate carboxylase, mitochondrial | 43.1 | 62.2 | 0.000 | Mitochondrion |
| 28 | *STAU2* | Double-stranded RNA-binding protein Staufen homolog 2 | 5.4 | 7.6 | 0.006 | Cytoplasm |
| 29 | *INA* | Alpha-internexin | 7.0 | 9.7 | 0.019 | Cytoplasm |

| 30 | *RABIF* | Guanine nucleotide exchange factor MSS4 | 12.0 | 16.6 | 0.007 | Cytosol |
| --- | --- | --- | --- | --- | --- | --- |
| 31 | *ARHGAP18* | Rho GTPase-activating protein 18 | 8.2 | 11.2 | 0.028 | Cytoplasm |
| 32 | *AK1* | Adenylate kinase isoenzyme 1 | 22.8 | 31.4 | 0.012 | Cytoplasm |
| 33 | *SH3KBP1* | SH3 domain-containing kinase-binding protein 1 | 19.5 | 26.7 | 0.006 | Cytoplasm |
| 34 | *DPYSL4* | Dihydropyrimidinase-related protein 4 | 34.2 | 46.7 | 0.008 | Cytoplasm |
| 35 | *TPD52L1* | Tumor protein D53 | 7.8 | 10.6 | 0.016 | Cytoplasm |
| 36 | *ARFIP1* | Arfaptin-1 | 7.9 | 10.6 | 0.036 | Golgi apparatus |
| 37 | *RAPH1* | Ras-associated and pleckstrin homology domains-containing  protein 1 | 11.9 | 16.1 | 0.004 | Cell membrane |
| 38 | *SETD7* | Histone-lysine N-methyltransferase SETD7 | 6.1 | 8.2 | 0.001 | Nucleus |
| 39 |  | Uncharacterized protein FLJ45252 | 6.0 | 8.0 | 0.038 | Unknown |
| 40 | *FGF2* | Fibroblast growth factor 2 | 25.1 | 33.4 | 0.037 | Secreted |
| 41 | *HEATR5A* | HEAT repeat-containing protein 5A | 5.3 | 7.0 | 0.024 | Cytosol |
| 42 | *HSPA2* | Heat shock-related 70 kDa protein 2 | 41.8 | 55.4 | 0.000 | Cytoplasm |
| 43 | *WDR81* | WD repeat-containing protein 81 | 9.2 | 12.0 | 0.013 | Endosome |
| 44 | *DUSP3* | Dual specificity protein phosphatase 3 | 20.3 | 26.4 | 0.003 | Nucleus |
| 45 | *MIF* | Macrophage migration inhibitory factor | 954.6 | 1239.8 | 0.037 | Secreted |
| 46 | *LTV1* | Protein LTV1 homolog | 6.2 | 8.0 | 0.004 | Cytoplasm |
| 47 | *VAC14* | Protein VAC14 homolog | 24.7 | 31.7 | 0.041 | Endosome |
| 48 | *UBA5* | Ubiquitin-like modifier-activating enzyme 5 | 9.1 | 11.6 | 0.004 | Cytoplasm |
| 49 | *ANXA3* | Annexin A3 | 38.9 | 49.5 | 0.033 | Cytoplasm |
| 50 | *ADK* | Adenosine kinase | 9.0 | 11.5 | 0.008 | Nucleus |
| 51 | *ABCF3* | ATP-binding cassette sub-family F member 3 | 11.1 | 14.0 | 0.004 | Cell membrane |
| 52 | *LUZP1* | Leucine zipper protein 1 | 5.2 | 6.6 | 0.030 | Cytoplasm |
| 53 | *AKR1C3* | Aldo-keto reductase family 1 member C3 | 1535.1 | 1936.8 | 0.008 | Cytoplasm |
| 54 | *EEF1A1;EEF1* | Elongation factor 1-alpha 1 | 1309.0 | 1648.6 | 0.011 | Cytoplasm |
| 55 | *AAK1* | AP2-associated protein kinase 1 | 12.9 | 16.1 | 0.036 | Cell membrane |
| 56 | *C1orf43* | Protein C1orf43 | 45.5 | 56.6 | 0.023 | Cell membrane |
| 57 | *DPYSL2* | Dihydropyrimidinase-related protein 2 | 175.0 | 217.4 | 0.000 | Cytoplasm |
| 58 | *BTBD11* | Ankyrin repeat and BTB/POZ domain-containing protein BTBD11 | 19.1 | 23.7 | 0.015 | Cell membrane |
| 59 | *NEU1* | Sialidase-1 | 16.8 | 20.8 | 0.037 | Lysosome |
| 60 | *AIDA* | Axin interactor, dorsalization-associated protein | 9.1 | 11.2 | 0.041 | Cytoplasm |
| **61** | ***PRDX1*** | **Peroxiredoxin-1** | **2037.3** | **2511.2** | **0.001** | **Cytoplasm** |

| 62 | *AP1S1* | AP-1 complex subunit sigma-1A | 10.7 | 13.2 | 0.040 | Golgi apparatus |
| --- | --- | --- | --- | --- | --- | --- |
| 63 | *VASP* | Vasodilator-stimulated phosphoprotein | 50.8 | 62.3 | 0.010 | Cytoplasm |
| 64 | *PGP* | Glycerol-3-phosphate phosphatase | 32.7 | 40.2 | 0.034 | Cytoplasm |
| 65 | *DUSP12* | Dual specificity protein phosphatase 12 | 9.8 | 12.0 | 0.013 | Nucleus |
| 66 | *GALM* | Galactose mutarotase | 19.4 | 23.7 | 0.012 | Cytoplasm |
| 67 | *PPM1A* | Protein phosphatase 1A | 9.1 | 11.2 | 0.034 | Nucleus |
| 68 | *CAPN2* | Calpain-2 catalytic subunit | 55.7 | 68.2 | 0.022 | Cytoplasm |
| 69 | *GGPS1* | Geranylgeranyl pyrophosphate synthase | 4.5 | 5.5 | 0.007 | Cytoplasm |
| 70 | *CRIP2* | Cysteine-rich protein 2 | 22.1 | 26.9 | 0.025 | Others |
| 71 | *ME1* | NADP-dependent malic enzyme | 37.9 | 46.0 | 0.004 | Cytoplasm |
| 72 | *ARIH1* | E3 ubiquitin-protein ligase ARIH1 | 10.2 | 12.4 | 0.040 | Cytoplasm |
| 73 | *CTPS1* | CTP synthase 1 | 32.3 | 39.1 | 0.006 | Cytoplasm |
| 74 | *BOLA2B* | BolA-like protein 2 | 48.4 | 58.7 | 0.0416 | Cytoplasm |
| 75 | *DNAJB4* | DnaJ homolog subfamily B member 4 | 15.8 | 19.1 | 0.031 | Cytoplasm |
| 76 | *NDRG3* | Protein NDRG3 | 14.8 | 17.9 | 0.011 | Cytoplasm |
| 77 | *UCHL3* | Ubiquitin carboxyl-terminal hydrolase isozyme L3 | 19.4 | 23.5 | 0.015 | Cytoplasm |
| 78 | *CSNK2A2* | Casein kinase II subunit alpha' | 21.6 | 26.1 | 0.044 | Nucleus |
| 79 | *CHMP4A* | Charged multivesicular body protein 4a | 18.7 | 22.5 | 0.025 | Cytoplasm |
| 80 | *PIP4K2C* | Phosphatidylinositol 5-phosphate 4-kinase type-2 gamma | 9.0 | 10.8 | 0.009 | Endoplasmic  recticulum |
| 81 | *C11orf54* | Ester hydrolase C11orf54 | 17.5 | 21.0 | 0.012 | Nucleus |
| 82 | *EEF1B2* | Elongation factor 1-beta | 135.3 | 162.5 | 0.038 | Cytoplasm |
| 83 | *PKM* | Pyruvate kinase PKM | 1844.9 | 2211.2 | 0.004 | Cytoplasm |
| 84 | *PPP1R12A* | Protein phosphatase 1 regulatory subunit 12A | 20.3 | 24.3 | 0.007 | Cytoplasm |
| 85 | *SNX1* | Sorting nexin-1 | 23.5 | 28.1 | 0.002 | Endoplasmic  recticulum |
| 86 | *GMPPA* | Mannose-1-phosphate guanyltransferase alpha | 7.1 | 8.5 | 0.013 | Cytoplasm |
| 87 | *TTC1* | Tetratricopeptide repeat protein 1 | 33.2 | 39.7 | 0.018 | Cytosol |
| 88 | *CES1* | Liver carboxylesterase 1 | 62.6 | 74.8 | 0.005 | Endoplasmic  recticulum |
| 89 | *ISYNA1* | Inositol-3-phosphate synthase 1 | 19.1 | 22.8 | 0.002 | Cytoplasm |
| 90 | *IRGQ* | Immunity-related GTPase family Q protein | 22.1 | 26.4 | 0.022 | Unknown |
| 91 | *NEK9* | Serine/threonine-protein kinase Nek9 | 20.4 | 24.3 | 0.003 | Cytoplasm |
| 92 | *KIFBP* | KIF-binding protein | 7.4 | 8.8 | 0.045 | Cytoplasm |

| 93 | *DNAJB1* | DnaJ homolog subfamily B member 1 | 33.8 | 40.1 | 0.027 | Cytoplasm |
| --- | --- | --- | --- | --- | --- | --- |
| 94 | *SGTA* | Small glutamine-rich tetratricopeptide repeat-containing protein  alpha | 17.8 | 21.1 | 0.008 | Cytoplasm |
| 95 | *GDI1* | Rab GDP dissociation inhibitor alpha | 84.2 | 99.7 | 0.011 | Cytoplasm |
| 96 | *ACAT2* | Acetyl-CoA acetyltransferase, cytosolic | 23.0 | 27.2 | 0.004 | Cytoplasm |
| 97 | *PITPNB* | Phosphatidylinositol transfer protein beta isoform | 10.4 | 12.3 | 0.043 | Golgi apparatus |
| 98 | *PPP2R5E* | Serine/threonine-protein phosphatase 2A 56 kDa regulatory  subunit epsilon isoform | 11.9 | 14.1 | 0.018 | Cytoplasm |
| 99 | *PLIN3* | Perilipin-3 | 31.5 | 37.2 | 0.001 | Others |
| 100 | *PDLIM1* | PDZ and LIM domain protein 1 | 179.5 | 211.8 | 0.020 | Cytoplasm |
| 101 | *PGAM1* | Phosphoglycerate mutase 1 | 541.0 | 637.7 | 0.004 | Cytoplasm |
| 102 | *LPP* | Lipoma-preferred partner | 40.6 | 47.8 | 0.004 | Nucleus |
| 103 | *TXNL1* | Thioredoxin-like protein 1 | 47.4 | 55.7 | 0.033 | Cytoplasm |
| 104 | *RPE* | Ribulose-phosphate 3-epimerase | 28.6 | 33.5 | 0.000 | Cytosol |
| 105 | *NRBP1* | Nuclear receptor-binding protein | 6.5 | 7.6 | 0.019 | Cytoplasm |
| 106 | *CIAO2B* | Cytosolic iron-sulfur assembly component 2B | 12.3 | 14.3 | 0.017 | Nucleus |
| 107 | *PPA1* | Inorganic pyrophosphatase | 49.0 | 57.3 | 0.041 | Cytoplasm |
| 108 | *TOMM34* | Mitochondrial import receptor subunit TOM34 | 20.7 | 24.2 | 0.030 | Cytoplasm |
| 109 | *PPME1* | Protein phosphatase methylesterase 1 | 22.9 | 26.7 | 0.039 | Nucleus |
| 110 | *SNAP25* | Synaptosomal-associated protein 25 | 11.4 | 13.3 | 0.038 | Cytoplasm |
| 111 | *SELENBP1* | Methanethiol oxidase | 28.2 | 32.8 | 0.010 | Nucleus |
| 112 | *PPID* | Peptidyl-prolyl cis-trans isomerase D | 231.7 | 269.0 | 0.040 | Cytoplasm |
| 113 | *VAT1* | Synaptic vesicle membrane protein VAT-1 homolog | 392.9 | 455.2 | 0.004 | Cytoplasm |
| 114 | *CCDC124* | Coiled-coil domain-containing protein 124 | 27.4 | 31.8 | 0.002 | Cytoplasm |
| 115 | *HMBS* | Porphobilinogen deaminase | 9.9 | 11.5 | 0.019 | Cytoplasm |
| 116 | *PPIA* | Peptidyl-prolyl cis-trans isomerase A | 1120.0 | 1294.3 | 0.034 | Cytoplasm |
| 117 | *TRMT6* | tRNA (adenine(58)-N(1))-methyltransferase non-catalytic subunit  TRM6 | 20.6 | 23.8 | 0.022 | Nucleus |
| 118 | *TKFC* | Triokinase/FMN cyclase | 22.1 | 25.5 | 0.020 | Cytosol |
| 119 | *ATIC* | Bifunctional purine biosynthesis protein ATIC | 221.3 | 255.2 | 0.008 | Cytoplasm |
| 120 | *SNX8* | Sorting nexin-8 | 9.1 | 10.5 | 0.029 | Endosome |
| 121 | *LUC7L* | Putative RNA-binding protein Luc7-like 1 | 19.5 | 22.4 | 1.95E-03 | Unknown |
| 122 | *USP15* | Ubiquitin carboxyl-terminal hydrolase 15 | 14.6 | 16.8 | 0.031 | Cytoplasm |

| 123 | *PNPLA6* | Patatin-like phospholipase domain-containing protein 6 | 13.4 | 15.4 | 0.020 | Endoplasmic  recticulum |
| --- | --- | --- | --- | --- | --- | --- |
| 124 | *PYCR3* | Pyrroline-5-carboxylate reductase 3 | 17.2 | 19.7 | 0.027 | Cytoplasm |
| 125 | *PPP1R12C* | Protein phosphatase 1 regulatory subunit 12C | 5.1 | 5.8 | 0.029 | Cytoplasm |
| 126 | *COPS8* | COP9 signalosome complex subunit 8 | 21.1 | 24.2 | 0.034 | Cytoplasm |
| 127 | *ABCF1* | ATP-binding cassette sub-family F member 1 | 25.9 | 29.6 | 0.017 | Cytoplasm |
| 128 | *GSPT1* | Eukaryotic peptide chain release factor GTP-binding subunit ERF3A | 38.7 | 44.3 | 0.040 | Cytoplasm |
| 129 | *PLS3* | Plastin-3 | 58.0 | 66.3 | 0.002 | Cytoplasm |
| 130 | *CCT4* | T-complex protein 1 subunit delta | 73.9 | 84.4 | 0.041 | Cytoplasm |
| 131 | *AHCY* | Adenosylhomocysteinase | 209.0 | 238.6 | 0.007 | Cytoplasm |
| 132 | *SPR* | Sepiapterin reductase | 51.8 | 59.2 | 0.015 | Cytoplasm |
| 133 | *OTUB1* | Ubiquitin thioesterase OTUB1 | 66.1 | 75.4 | 0.031 | Cytoplasm |
| 134 | *ZC3H15* | Zinc finger CCCH domain-containing protein 15 | 23.4 | 26.6 | 0.035 | Cytoplasm |
| 135 | *ILK* | Integrin-linked protein kinase | 33.5 | 38.0 | 0.026 | Others |
| 136 | *ADSL* | Adenylosuccinate lyase | 15.9 | 17.9 | 0.0080 | Cytosol |
| 137 | *FASN* | Fatty acid synthase | 253.2 | 286.5 | 0.008 | Cytoplasm |
| 138 | *SKP1* | S-phase kinase-associated protein 1 | 44.6 | 50.4 | 0.041 | Cytoplasm |
| 139 | *AHNAK2* | Protein AHNAK2 | 54.6 | 61.6 | 0.003 | Nucleus |
| 140 | *AIP* | AH receptor-interacting protein | 22.0 | 24.8 | 0.006 | Cytoplasm |
| 141 | *BAG6* | Large proline-rich protein BAG6 | 31.6 | 35.5 | 0.043 | Cytoplasm |
| 142 | *UGDH* | UDP-glucose 6-dehydrogenase | 768.6 | 862.8 | 0.001 | Cytosol |
| 143 | *SMS* | Spermine synthase | 25.0 | 28.0 | 0.034 | Cytosol |
| 144 | *ALDH1B1* | Aldehyde dehydrogenase X, mitochondrial | 60.2 | 67.4 | 0.004 | Mitochondrion |
| 145 | *KIF2A* | Kinesin-like protein KIF2A | 15.0 | 16.8 | 0.007 | Cytoplasm |
| 146 | *USP14* | Ubiquitin carboxyl-terminal hydrolase 14 | 34.5 | 38.6 | 0.044 | Cytoplasm |
| 147 | *UBE2O* | (E3-independent) E2 ubiquitin-conjugating enzyme | 9.7 | 10.8 | 0.012 | Cytoplasm |
| 148 | *IGBP1* | Immunoglobulin-binding protein 1 | 31.1 | 34.7 | 0.004 | Cytoplasm |
| 149 | *TNS3* | Tensin-3 | 50.5 | 56.4 | 0.038 | Others |
| 150 | *PRDX5* | Peroxiredoxin-5, mitochondrial | 248.9 | 277.9 | 0.039 | Mitochondrion |
| 151 | *HSPB1* | Heat shock protein beta-1 | 768.3 | 856.3 | 3.78E-03 | Cytoplasm |
| 152 | *REXO2* | Oligoribonuclease, mitochondrial | 28.8 | 32.1 | 0.032 | Mitochondrion |
| 153 | *CASK* | Peripheral plasma membrane protein CASK | 13.2 | 14.7 | 0.024 | Nucleus |
| 154 | *PSMC3* | 26S proteasome regulatory subunit 6A | 57.3 | 63.7 | 0.007 | Cytoplasm |

| 155 | *ACLY* | ATP-citrate synthase | 263.1 | 292.1 | 0.030 | Cytoplasm |
| --- | --- | --- | --- | --- | --- | --- |
| 156 | *ATP6V1A* | V-type proton ATPase catalytic subunit A | 53.4 | 59.3 | 0.008 | Cytoplasm |
| 157 | *COPS3* | COP9 signalosome complex subunit 3 | 32.5 | 36.0 | 0.020 | Cytoplasm |
| 158 | *GPI* | Glucose-6-phosphate isomerase | 544.6 | 602.6 | 4.25E-02 | Cytoplasm |
| 159 | *DNAJA1* | DnaJ homolog subfamily A member 1 | 43.2 | 47.7 | 0.018 | Cell membrane |
| 160 | *LRRC47* | Leucine-rich repeat-containing protein 47 | 73.7 | 81.3 | 0.010 | Unknown |
| 161 | *NAP1L1* | Nucleosome assembly protein 1-like 1 | 73.4 | 81.0 | 0.023 | Cytoplasm |
| 162 | *ANKHD1* | Ankyrin repeat and KH domain-containing protein 1 | 10.3 | 11.3 | 0.026 | Cytoplasm |
| 163 | *GCN1* | eIF-2-alpha kinase activator GCN1 | 65.7 | 71.8 | 0.005 | Cytoplasm |
| 164 | *ATP6V1E1* | V-type proton ATPase subunit E 1 | 43.8 | 47.9 | 0.033 | Others |
| 165 | *PPM1G* | Protein phosphatase 1G | 30.8 | 33.6 | 0.032 | Cytoplasm |
| 166 | *ALDH9A1* | 4-trimethylaminobutyraldehyde dehydrogenase | 22.4 | 24.4 | 0.0147 | Cytoplasm |
| 167 | *MARS1* | Methionine--tRNA ligase, cytoplasmic | 106.8 | 115.9 | 0.013 | Cytoplasm |
| 168 | *DHX30* | ATP-dependent RNA helicase DHX30 | 19.1 | 20.7 | 0.035 | Cytoplasm |
| 169 | *HK1* | Hexokinase-1 | 64.7 | 69.9 | 0.034 | Mitochondrion |
| 170 | *SEC31A* | Protein transport protein Sec31A | 53.1 | 57.2 | 0.031 | Cytoplasm |
| 171 | *SEC23A* | Protein transport protein Sec23A | 49.6 | 53.3 | 0.029 | Cytoplasm |
| 172 | *UFD1* | Ubiquitin recognition factor in ER-associated degradation protein 1 | 77.0 | 82.4 | 0.044 | Nucleus |
| 173 | *SEPTIN2* | Septin-2 | 52.5 | 56.1 | 0.044 | Cytoplasm |
| 174 | *QARS1* | Glutamine--tRNA ligase | 42.7 | 45.6 | 0.010 | Cytoplasm |
| 175 | *PSMD4* | 26S proteasome non-ATPase regulatory subunit 4 | 40.3 | 42.8 | 0.027 | Cytoplasm |
| 176 | *PPP2R1A* | Serine/threonine-protein phosphatase 2A 65 kDa regulatory  subunit A alpha isoform | 61.0 | 64.7 | 0.0277 | Cytoplasm |
| 177 | *ACACA* | Acetyl-CoA carboxylase 1 | 41.2 | 43.3 | 0.027 | Cytoplasm |
| **Downregulation** | | | | | | |
| No. | Genes | Proteins | Control | Radon | *P* -value | Location |
| 1 | *UNC93B1* | Protein unc-93 homolog B1 | 7.7 | 1.2 | 0.014 | Endoplasmic  recticulum |
| 2 | *TMEM256* | Transmembrane protein 256 | 14.8 | 2.5 | 0.015 | Cell membrane |
| 3 | *CNEP1R1* | Nuclear envelope phosphatase-regulatory subunit 1 | 1.6 | 0.3 | 0.027 | Nucleus |
| 4 | *GUSB* | Beta-glucuronidase | 3.3 | 0.7 | 0.041 | Lysosome |
| 5 | *DNAJC17* | DnaJ homolog subfamily C member 17 | 9.4 | 2.8 | 0.016 | Cytoplasm |
| 6 | *GABRB3* | Gamma-aminobutyric acid receptor subunit beta-3 | 7.2 | 2.7 | 0.019 | Cell membrane |

| 7 | *NOP53* | Ribosome biogenesis protein NOP53 | 11.9 | 7.5 | 0.024 | Nucleus |
| --- | --- | --- | --- | --- | --- | --- |
| 8 | *CLDN2* | Claudin-2 | 6.2 | 4.1 | 0.002 | Cell membrane |
| 9 | *LSM6* | U6 snRNA-associated Sm-like protein LSm6 | 6.3 | 4.3 | 0.028 | Cytoplasm |
| 10 | *DCTN3* | Dynactin subunit 3 | 11.5 | 8.3 | 0.018 | Cytoplasm |
| 11 | *CREG1* | Protein CREG1 | 61.6 | 44.7 | 0.024 | Secreted |
| 12 | *SERPINE2* | Glia-derived nexin | 19.3 | 14.0 | 0.007 | Secreted |
| 13 | *NFU1* | NFU1 iron-sulfur cluster scaffold homolog, mitochondrial | 14.5 | 10.6 | 0.007 | Mitochondrion |
| 14 | *ALDH3B1* | Aldehyde dehydrogenase family 3 member B1 | 52.8 | 39.0 | 0.006 | Cell membrane |
| 15 | *TPM1* | Tropomyosin alpha-1 chain | 24.4 | 18.1 | 0.011 | Cytoplasm |
| 16 | *HMG20A* | High mobility group protein 20A | 9.9 | 7.4 | 0.031 | Nucleus |
| 17 | *A2M* | Alpha-2-macroglobulin | 10.9 | 8.3 | 0.009 | Secreted |
| 18 | *CTSC* | Dipeptidyl peptidase 1 | 23.4 | 18.1 | 0.010 | Lysosome |
| 19 | *WDR26* | WD repeat-containing protein 26 | 8.9 | 6.9 | 0.030 | Cytoplasm |
| 20 | *CCNY* | Cyclin-Y | 3.7 | 2.9 | 0.038 | Cell membrane |
| 21 | *LMNB1* | Lamin-B1 | 65.6 | 51.4 | 0.006 | Nucleus |
| 22 | *LBR* | Delta(14)-sterol reductase LBR | 18.6 | 14.6 | 0.005 | Nucleus |
| 23 | *TMPO* | Lamina-associated polypeptide 2, isoforms beta/gamma | 66.5 | 52.2 | 0.003 | Nucleus |
| 24 | *ALDH1L2* | Mitochondrial 10-formyltetrahydrofolate dehydrogenase | 32.3 | 25.6 | 0.030 | Mitochondrion |
| 25 | *CPS1* | Carbamoyl-phosphate synthase [ammonia], mitochondrial | 93.3 | 73.9 | 0.036 | Mitochondrion |
| 26 | *SEC63* | Translocation protein SEC63 homolog | 10.5 | 8.4 | 0.004 | Endoplasmic  recticulum |
| 27 | *SCARB1* | Scavenger receptor class B member 1 | 28.0 | 22.3 | 0.014 | Cell membrane |
| 28 | *CISD3* | CDGSH iron-sulfur domain-containing protein 3, mitochondrial | 13.7 | 11.0 | 0.040 | Mitochondrion |
| 29 | *NECTIN2* | Nectin-2 | 17.1 | 13.7 | 0.009 | Cell membrane |
| 30 | *SURF6* | Surfeit locus protein 6 | 20.6 | 16.5 | 0.011 | Nucleus |
| 31 | *CEBPB* | CCAAT/enhancer-binding protein beta | 22.6 | 18.2 | 0.002 | Nucleus |
| 32 | *TBL3* | Transducin beta-like protein 3 | 31.1 | 25.0 | 0.031 | Nucleus |
| 33 | *ECI1* | Enoyl-CoA delta isomerase 1, mitochondrial | 142.6 | 115.0 | 0.004 | Mitochondrion |
| 34 | *SLC12A2* | Solute carrier family 12 member 2 | 39.1 | 31.7 | 0.012 | Cell membrane |
| 35 | *RBM10* | RNA-binding protein 10 | 12.6 | 10.2 | 0.021 | Nucleus |
| 36 | *BSG* | Basigin | 87.5 | 71.0 | 0.017 | Cell membrane |
| 37 | *TMX4* | Thioredoxin-related transmembrane protein 4 | 8.4 | 6.8 | 0.021 | Nucleus |
| 38 | *CTNNB1* | Catenin beta-1 | 24.4 | 19.8 | 0.024 | Cytoplasm |

| 39 | *NOC2L* | Nucleolar complex protein 2 homolog | 20.9 | 17.0 | 0.043 | Nucleus |
| --- | --- | --- | --- | --- | --- | --- |
| 40 | *DPP7* | Dipeptidyl peptidase 2 | 19.1 | 15.6 | 0.038 | Lysosome |
| 41 | *NHP2* | H/ACA ribonucleoprotein complex subunit 2 | 20.4 | 16.7 | 0.031 | Nucleus |
| 42 | *DNAJC3* | DnaJ homolog subfamily C member 3 | 12.5 | 10.3 | 0.033 | Endoplasmic  recticulum |
| 43 | *NTHL1* | Endonuclease III-like protein 1 | 10.2 | 8.4 | 0.012 | Nucleus |
| 44 | *NONO* | Non-POU domain-containing octamer-binding protein | 228.2 | 187.5 | 0.026 | Nucleus |
| 45 | *NUP54* | Nucleoporin p54 | 20.0 | 16.4 | 0.018 | Nucleus |
| 46 | *NOLC1* | Nucleolar and coiled-body phosphoprotein 1 | 44.8 | 37.0 | 0.018 | Nucleus |
| 47 | *H1-10* | Histone H1.10 | 24.7 | 20.4 | 0.025 | Nucleus |
| 48 | *PRPF19* | Pre-mRNA-processing factor 19 | 52.9 | 43.8 | 0.039 | Nucleus |
| 49 | *FTL* | Ferritin light chain | 58.4 | 48.4 | 0.018 | Cytoplasm |
| 50 | *HLA-A* | HLA class I histocompatibility antigen, A alpha chain | 28.0 | 23.3 | 0.005 | Cell membrane |
| 51 | *CANX* | Calnexin | 359.9 | 298.7 | 0.034 | Endoplasmic  recticulum |
| 52 | *GAR1* | H/ACA ribonucleoprotein complex subunit 1 | 85.7 | 71.6 | 0.030 | Nucleus |
| 53 | *MRPL14* | 39S ribosomal protein L14, mitochondrial | 16.5 | 13.8 | 0.016 | Mitochondrion |
| 54 | *TMX3* | Protein disulfide-isomerase TMX3 | 21.5 | 18.0 | 0.025 | Endoplasmic  recticulum |
| 55 | *CD81* | CD81 antigen | 37.6 | 31.6 | 0.014 | Cell membrane |
| 56 | *NRP1* | Neuropilin-1 | 24.2 | 20.3 | 0.006 | Secreted |
| 57 | *PCK2* | Phosphoenolpyruvate carboxykinase [GTP], mitochondrial | 85.8 | 72.3 | 0.016 | Mitochondrion |
| 58 | *ERO1A* | ERO1-like protein alpha | 50.6 | 42.7 | 0.028 | Endoplasmic  recticulum |
| 59 | *PRPF38A* | Pre-mRNA-splicing factor 38A | 22.5 | 19.0 | 0.001 | Nucleus |
| 60 | *RBBP5* | Retinoblastoma-binding protein 5 | 10.8 | 9.2 | 0.016 | Nucleus |
| 61 | *WDR18* | WD repeat-containing protein 18 | 51.6 | 43.7 | 0.026 | Nucleus |
| 62 | *MATR3* | Matrin-3 | 100.0 | 84.8 | 0.040 | Nucleus |
| 63 | *LMNB2* | Lamin-B2 | 31.7 | 26.9 | 0.024 | Nucleus |
| 64 | *SF3A3* | Splicing factor 3A subunit 3 | 31.5 | 26.8 | 0.002 | Nucleus |
| 65 | *SHMT2* | Serine hydroxymethyltransferase, mitochondrial | 209.3 | 178.1 | 0.004 | Mitochondrion |
| 66 | *GLRX5* | Glutaredoxin-related protein 5, mitochondrial | 15.1 | 12.9 | 0.042 | Mitochondrion |
| 67 | *ACP2* | Lysosomal acid phosphatase | 21.4 | 18.3 | 0.030 | Lysosome |
| 68 | *NPM1* | Nucleophosmin | 2179.1 | 1864.1 | 0.005 | Nucleus |

| 69 | *FKBP10* | Peptidyl-prolyl cis-trans isomerase FKBP10 | 22.1 | 18.9 | 0.009 | Endoplasmic  recticulum |
| --- | --- | --- | --- | --- | --- | --- |
| 70 | *HEXA* | Beta-hexosaminidase subunit alpha | 26.2 | 22.4 | 0.017 | Lysosome |
| 71 | *OS9* | Protein OS-9 | 19.3 | 16.6 | 0.037 | Endoplasmic  recticulum |
| 72 | *IGF2R* | Cation-independent mannose-6-phosphate receptor | 40.2 | 34.8 | 0.009 | Golgi apparatus |
| 73 | *ARSL* | Arylsulfatase L | 36.1 | 31.2 | 0.029 | Golgi apparatus |
| 74 | *MRPS11* | 28S ribosomal protein S11, mitochondrial | 41.0 | 35.5 | 0.004 | Mitochondrion |
| 75 | *SNU13* | NHP2-like protein 1 | 70.5 | 61.1 | 0.023 | Nucleus |
| 76 | *PHB2* | Prohibitin-2 | 481.9 | 417.8 | 0.016 | Mitochondrion |
| 77 | *LONP1* | Lon protease homolog, mitochondrial | 89.8 | 77.8 | 0.012 | Mitochondrion |
| 78 | *HDAC2* | Histone deacetylase 2 | 40.1 | 34.8 | 0.021 | Nucleus |
| 79 | *TJP1* | Tight junction protein ZO-1 | 26.2 | 22.8 | 0.034 | Cell membrane |
| 80 | *TOP1* | DNA topoisomerase 1 | 60.4 | 52.6 | 0.018 | Nucleus |
| 81 | *SPCS2* | Signal peptidase complex subunit 2 | 39.3 | 34.3 | 0.045 | Endoplasmic  recticulum |
| 82 | *HNRNPL* | Heterogeneous nuclear ribonucleoprotein L | 131.7 | 115.0 | 0.043 | Nucleus |
| 83 | *SIN3A* | Paired amphipathic helix protein Sin3a | 9.6 | 8.4 | 0.043 | Nucleus |
| 84 | *TBL2* | Transducin beta-like protein 2 | 18.3 | 16.0 | 0.032 | Endoplasmic  recticulum |
| 85 | *ATP2A2* | Sarcoplasmic/endoplasmic reticulum calcium ATPase 2 | 238.9 | 210.3 | 0.025 | Endoplasmic  recticulum |
| 86 | *GNAI2* | Guanine nucleotide-binding protein G(i) subunit alpha-2 | 16.6 | 14.6 | 0.008 | Cytoplasm |
| 87 | *RCC1* | Regulator of chromosome condensation | 40.3 | 35.5 | 0.023 | Nucleus |
| 88 | *IGF2BP3* | Insulin-like growth factor 2 mRNA-binding protein 3 | 24.7 | 21.7 | 0.017 | Nucleus |
| 89 | *PHB1* | Prohibitin 1 | 419.1 | 369.6 | 0.007 | Mitochondrion |
| 90 | *PIGK* | GPI-anchor transamidase | 20.8 | 18.3 | 0.027 | Endoplasmic  recticulum |
| 91 | *GAA* | Lysosomal alpha-glucosidase | 77.1 | 68.0 | 0.027 | Lysosome |
| 92 | *SSR4* | Translocon-associated protein subunit delta | 83.0 | 73.4 | 0.013 | Endoplasmic  recticulum |
| 93 | *MRTO4* | mRNA turnover protein 4 homolog | 31.7 | 28.1 | 0.037 | Nucleus |
| 94 | *AUP1* | Lipid droplet-regulating VLDL assembly factor AUP1 | 18.9 | 16.8 | 0.039 | Endoplasmic  recticulum |

| 95 | *ITGA3* | Integrin alpha-3 | 114.5 | 101.6 | 0.020 | Cell membrane |
| --- | --- | --- | --- | --- | --- | --- |
| 96 | *DSG2* | Desmoglein-2 | 56.3 | 50.0 | 0.017 | Cell membrane |
| 97 | *PALS2* | Protein PALS2 | 18.0 | 16.0 | 0.040 | Cell membrane |
| 98 | *FUBP3* | Far upstream element-binding protein 3 | 68.8 | 61.1 | 0.036 | Nucleus |
| 99 | *RBM27* | RNA-binding protein 27 | 9.4 | 8.3 | 0.027 | Cytoplasm |
| 100 | *ISOC2* | Isochorismatase domain-containing protein 2 | 34.7 | 30.8 | 0.039 | Cytoplasm |
| 101 | *RCC2* | Protein RCC2 | 33.5 | 29.8 | 0.022 | Nucleus |
| 102 | *CD109* | CD109 antigen | 21.0 | 18.7 | 0.011 | Cell membrane |
| 103 | *SNRPB* | Small nuclear ribonucleoprotein-associated proteins B and B' | 185.6 | 165.6 | 0.028 | Cytoplasm |
| 104 | *ACOX1* | Peroxisomal acyl-coenzyme A oxidase 1 | 28.4 | 25.4 | 0.021 | Peroxisome |
| 105 | *FLOT1* | Flotillin-1 | 100.8 | 90.2 | 0.030 | Cell membrane |
| 106 | *RBBP4* | Histone-binding protein RBBP4 | 21.7 | 19.4 | 0.028 | Nucleus |
| 107 | *RRAS* | Ras-related protein R-Ras | 22.2 | 20.0 | 0.021 | Cell membrane |
| 108 | *TPR* | Nucleoprotein TPR | 32.6 | 29.4 | 0.040 | Nucleus |
| **109** | ***ALDH2*** | **Aldehyde dehydrogenase, mitochondrial** | **112.5** | **101.3** | **0.010** | **Mitochondrion** |
| 110 | *SF3A1* | Splicing factor 3A subunit 1 | 20.6 | 18.5 | 0.010 | Nucleus |
| 111 | *EIF4A3* | Eukaryotic initiation factor 4A-III | 42.3 | 38.1 | 0.026 | Nucleus |
| 112 | *HEXB* | Beta-hexosaminidase subunit beta | 57.4 | 51.8 | 0.042 | Lysosome |
| 113 | *PUM3* | Pumilio homolog 3 | 40.8 | 37.3 | 0.024 | Nucleus |
| 114 | *AP2A2* | AP-2 complex subunit alpha-2 | 59.3 | 54.3 | 0.005 | Cell membrane |
| 115 | *TECR* | Very-long-chain enoyl-CoA reductase | 14.6 | 13.5 | 0.027 | Endoplasmic  recticulum |
| 116 | *ITGAV* | Integrin alpha-V | 32.0 | 29.8 | 0.034 | Cell membrane |
| 117 | *ACO2* | Aconitate hydratase, mitochondrial | 45.6 | 42.4 | 0.037 | Cell membrane |
| 118 | *SLC25A24* | Calcium-binding mitochondrial carrier protein SCaMC-1 | 34.2 | 31.9 | 0.032 | Mitochondrion |
| 119 | *MRPL47* | 39S ribosomal protein L47, mitochondrial | 19.3 | 18.1 | 0.036 | Mitochondrion |
| 120 | *RAB5C* | Ras-related protein Rab-5C | 48.4 | 45.4 | 0.032 | Cell membrane |
| 121 | *DDX42* | ATP-dependent RNA helicase DDX42 | 38.4 | 36.5 | 0.035 | Cytoplasm |
