# Supplementary figures and images for: Impact of intermittent high-dose radon exposures on lung epithelial cells: proteomic analysis and biomarker identification
Source: J Radiat Res. 2025 Mar 15;66(2):107–14. doi: 10.1093/jrr/rraf010 (PMC11932336; doi:10.1093/jrr/rraf010)

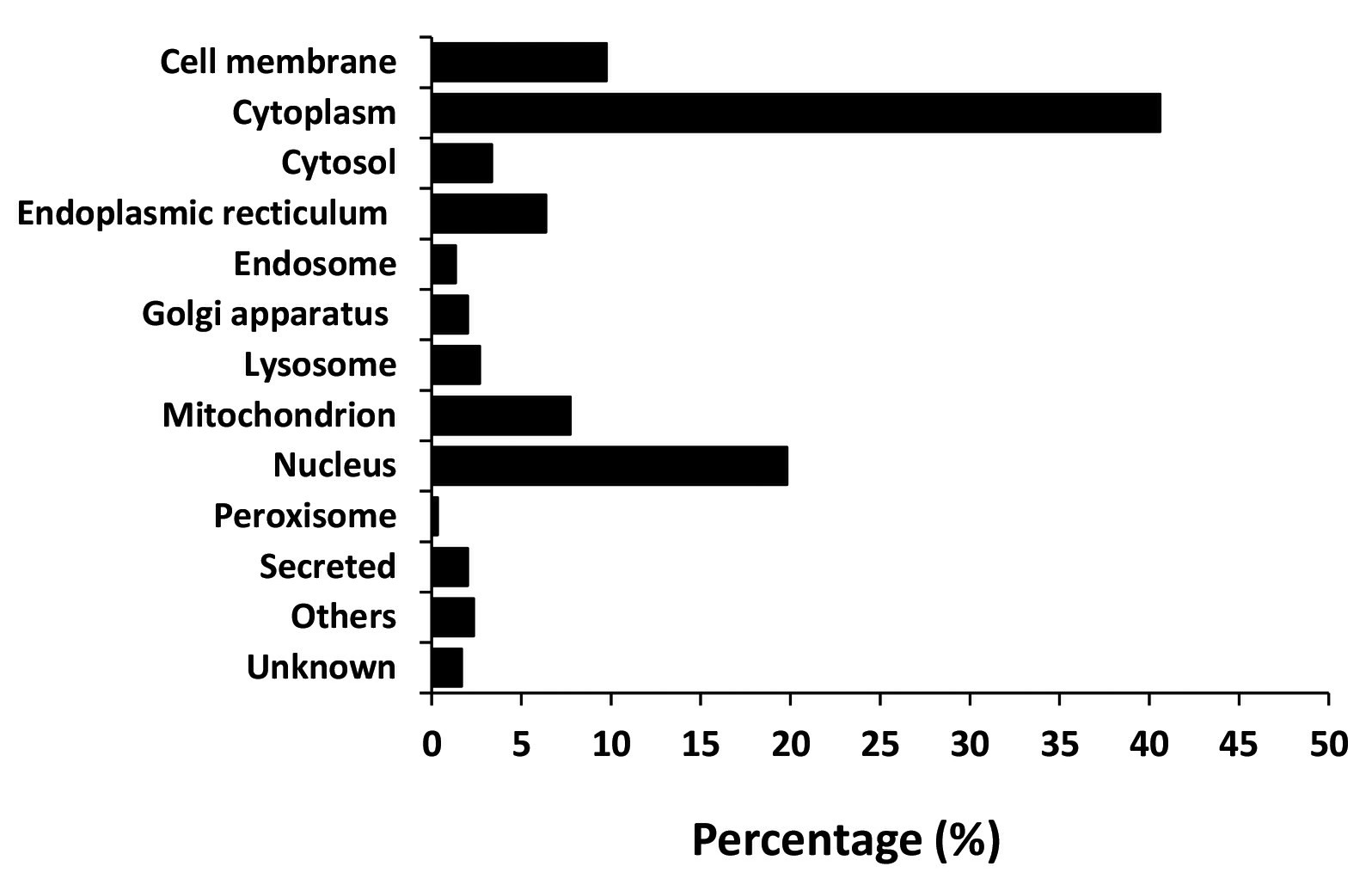

Supplement: Fig_S1_rraf010 [file fig_s1_rraf010.jpeg]
